# Editorial for the Special Issue on Advanced Interconnect and Packaging, 2nd Edition

**DOI:** 10.3390/mi15050643

**Published:** 2024-05-11

**Authors:** Dawei Wang, Wensheng Zhao

**Affiliations:** School of Electronics and Information and School of Integrated Circuit Science and Engineering, Hangzhou Dianzi University, Hangzhou 310018, China; davidw.zoeq@hdu.edu.cn

Interconnect and packaging technologies are crucial aspects of modern electronics, and they are essential to achieve high performance, miniaturization and low power consumption of electronic equipment [1]. With the rapid development of semiconductor processes, interconnect and packaging technologies continue to evolve to meet growing market demands. Packaging technology is responsible for protecting the chip and providing a suitable environment where it can work properly [2]. As the core part of packaging, interconnect technology is responsible for connecting various components inside the chip to each other and for exchanging signals and power with the outside systems. Interconnect technologies continue to evolve as the speed, density and functionality of semiconductor products increase. For example, flip bonding, through-silicon via (TSV) and hybrid bonding are widely used [3,4,5,6].

Considering the important roles that the advanced interconnect and packaging technologies play in advancing the semiconductor industry, it is necessary to provide a platform for the cutting-edge research and developments in this field. This Special Issue seeks to showcase papers on the developments in advanced interconnect and packaging technologies. In particular, Gulsaran et al. proposed a built-in packaging method for two-terminal devices in *Contribution* 1. The proposed method can increase the overlap area and reduce contact resistance, thereby providing a promising method for sensing applications, wireless power transfer, energy harvesting and solar rectennas. 

In the MEMS field, special functional coating was usually used to prolong service life. In *Contribution* 2, Zhao et al. employed the ultrasonic assisted electrochemical potential activation method to improve bonding strength. By utilizing the indentation method, bonding strength was measured, and the results demonstrated that the oxygen content of the substrate surface can be removed; moreover, the dislocation density of the electroplating Ni coating was reduced. The proposed method is beneficial for improving the interfacial bonding strength of MEMS devices [7]. 

Due to a low dielectric loss, a glass interposer was used for high-performance high-frequency applications. The accurate characterization of through-glass via (TGV) interconnects is critical for their applications [8,9]. However, non-coplanar ends would pose challenges to the testing process. To solve this problem, Liu et al. developed an accurate extraction method of the S parameters of a TGV interconnect using the transmission matrix in *Contribution* 3. This method can handle a diverse range of vertical interconnects, thereby providing an efficient measurement method up to 40 GHz. 

Further, signal and power integrity analyses of high-speed interconnects were conducted. *Contribution* 4 proposed a novel statistical approach for co-design signal and power integrity with the consideration of non-linear power/ground noise. This approach can be used to estimate the statistical eye diagram. Moreover, within this study, prediction accuracy was validated by comparing the results with HSPICE simulated results [10,11]. Then, in *Contribution* 5, Sun and Xu studied the crosstalk effect in high-speed package interconnects. They pointed out that delay-insensitive coding can reduce crosstalk peaks, thereby reducing wiring spacing. 

Integrated passive device (IPD) technology can package passive components together with active circuits. However, directional couplers, one of the most widely used components, have had few successful attempts based on IPD technology [12]. By utilizing defect ground structure and wiggly coupled lines, *Contribution* 6 presented the design and implementation of two 3 dB directional couplers based on silicon IPD technology. The results demonstrated that the proposed couplers can be used in low-cost, high-performance system-on-package front-end circuits. 

Finally, reliability issues were discussed as these problems were exacerbated with the continuous improvement of miniaturization and increased package density [13]. *Contribution* 7 investigated the electromigration reliability of Au-Al and OPM wire-bonded contacts using the resistance monitoring method. The results indicated that the homogeneous metal contacts can improve the immunity to electromigration.

The articles published in this Special Issue present important advancements in the field of interconnect and packaging technologies. I would like to thank all the authors who provided insights and shared their viewpoints and solutions. I also would like to thank the editors and reviewers who helped to improve the papers published in this Special Issue, especially Mr. Dikies Zhang and Ms. Aria Zeng from the publishing offices of *Micromachines*. However, it is worth noting that several challenges and obstacles remain to be addressed in the future. Nevertheless, we hope that the articles published in this Special Issue will be interesting and inspiring for their readers.

## List of Contributions

1. Gulsaran, A.; Azer, B.B.; Ozyigit, D.; Saritas, R.; Abdel-Rahman, E.; Yavuz, M. Built-in packaging for two-terminal devices. *Micromachines* **2023**, *14*, 1473. https://doi.org/10.3390/mi14071473.
2. Zhao, Z.; Huo, G.; Li, H. Solving the bonding problem of the Ni thin coating with the ultrasonic assisted electrochemical potential activation method, *Micromachines* **2023**, *14*, 34. https://doi.org/10.3390/mi14010034.
3. Liu, J.; Zhang, J.; Gao, L.; Chen, H. Extracting and analyzing the S-parameters of vertical interconnection structures in 3D glass packaging. *Micromachines* **2023**, *14*, 803. https://doi.org/10.3390/mi14040803.
4. Kim, Y. A statistical approach for signal and power integrity co-design in high-speed interconnects considering non-linear power/ground noise and bit-patterns. *Micromachines* **2023**, *14*, 1654. https://doi.org/10.3390/mi14091654.
5. Su, B.; Xu, Z. Crosstalk analysis of delay-insensitive code in high-speed package interconnects. *Micromachines* **2023**, *14*, 1033. https://doi.org/10.3390/mi14051033
6. Xu, M.; Su, J.; Wang, R.; Lin, Z.; Xie, W.; Liu, J. Design and implementation of broadband hybrid 3-dB couplers with silicon-based IPD technology. *Micromachines* **2023**, *14*, 932. https://doi.org/10.3390/mi14050932.
7. Li, X.; Gao, L.; Ni, T.; Zhou, J.; Li, X.; Li, Y.; Xu, L.; Wang, R.; Zeng, C.; Li, B.; et al. Analysis of degradation of electromigration reliability of Au-Al and OPM wire bonded contacts at 250 ℃ using resistance monitoring method. *Micromachines* **2023**, *14*, 640. https://doi.org/10.3390/mi14030640.
